# miR-99a-5p: A Potential New Therapy for Atherosclerosis by Targeting mTOR and Then Inhibiting NLRP3 Inflammasome Activation and Promoting Macrophage Autophagy

**DOI:** 10.1155/2022/7172583

**Published:** 2022-08-05

**Authors:** Gang Wang, Shu-Yan Jing, Gang Liu, Xue-Jia Guo, Wei Zhao, Xin-Le Jia, Ming-Qi Zheng, Wen-Yun Tan

**Affiliations:** ^1^Department of Cardiovascular Medicine, 980 Hospital of PLA Joint Logistics Support Forces, China; ^2^Department of Radiation and Nuclear Medicine, The First Hospital of Hebei Medical University, China; ^3^Department of Cardiovascular Medicine, The First Hospital of Hebei Medical University, China; ^4^Department of Ultrasound, The First Hospital of Hebei Medical University, China; ^5^Hebei Key Laboratory of Heart and Metabolism, China

## Abstract

**Objective:**

MicroRNAs have been revealed to be involved in the development of atherosclerosis. The present study is aimed at exploring the potential of miR-99a-5p as a therapy for atherosclerosis. We suspected that miR-99a-5p might inhibit NLRP3 inflammasome activation and promote macrophage autophagy via constraining mTOR, therefore, alleviating atherosclerosis.

**Methods:**

The cell viability in ox-LDL-induced THP-1 macrophages was assessed by CCK-8 assay. Bioinformatic analysis was used to predict the target genes of miR-99a-5p. The binding between miR-99a-5p and mTOR was confirmed by luciferase reporter assay. In vivo, a high-fat-diet-induced atherosclerosis model was established in apolipoprotein E knockout mice. Hematoxylin-eosin, oil red O, and Sirius red staining were performed for the determination of atherosclerotic lesions. MTOR and associated protein levels were detected by Western blot analysis.

**Results:**

miR-99a-5p inhibited NLRP3 inflammasome activation and promoted macrophage autophagy by targeting mTOR. Enforced miR-99a-5p significantly reduced the levels of inflammasome complex and inflammatory cytokines. Furthermore, miR-99a-5p overexpression inhibited the expression of mTOR, whereas mTOR overexpression reversed the trend of the above behaviors. In vivo, the specific overexpression of miR-99a-5p significantly reduced atherosclerotic lesions, accompanied by a significant downregulation of autophagy marker CD68 protein expression.

**Conclusion:**

We demonstrated for the first time that miR-99a-5p may be considered a therapy for atherosclerosis. The present study has revealed that miR-99a-5p might inhibit NLRP3 inflammasome activation and promote macrophage autophagy by targeting mTOR, therefore, alleviating atherosclerosis.

## 1. Introduction

Atherosclerosis (AS) now accounts for the majority of mortality worldwide and is characterized by chronic inflammatory infiltration, endothelial cell dysfunction, and lipid accumulation in the artery wall [1–3]. This global spread urgently needs to understand the genesis of this malady, advance in its management, and develop prospects for reducing its burden [3]. Over the last few decades, although traditional statins as powerful tools facilitated unprecedented reductions in low-density lipoprotein cholesterol (LDL-C), a considerable residual risk of cardiovascular events remained in these patients [1, 4, 5]. Recent studies have shown that the cells were induced by activated NOD-, LRR-, and pyrin domain-containing protein 3 (NLRP3) to secrete proinflammatory factors (1L-1*β*, TNF, etc.), therefore, triggering inflammatory responses and driving AS [1–3, 6, 7]. In parallel, AS is associated with inflammatory cells (mainly including macrophages and endothelial cells) at all stages [4, 8]. Moreover, previous studies have also indicated that enhanced autophagy in macrophages could restrain activation of NLRP3 inflammasome and then reduce the inflammatory response, thereby, inhibiting the development of AS progression [9, 10]. Therefore, increasing studies have focused on enhancing the autophagy of macrophages or inhibiting the activation of the inflammasome, which has emerged as the novel promising mode of therapy to improve and complement the current approaches [1, 4, 11–13]. Although related drugs or inhibitors have already shown initial activity, the therapeutic effect is limited, and the specific pathological mechanism is not fully elucidated [14–16]. Therefore, novel complementary therapeutic approaches will likely be necessary for treating disease states to break the deadlock.

Over the past decades, microRNAs (miRNAs) have emerged as evolutionarily conserved noncoding small RNAs that regulated apoptosis, proliferation, and differentiation, and served as important regulators and “fine-tuners” of a range of pathophysiological cellular effects and molecular signaling pathways involved in AS [17]. Recent studies have implicated that miRNA could regulate the behavior of macrophages and the inflammatory response mediated by NLRP3 in the progression of AS [18, 19]. The miRNAs might support better management of AS due to their potential applications as novel therapeutics, clinical biomarkers, and diagnostic or prognostic indicators [20, 21]. So far, systemic administration of either anti-miRNA delivery or the introduction of synthetic miRNAs (known as miRNA mimic) may thereby offer an attractive therapeutic approach to alleviate AS and facilitate the management of its complications [20]. A recent study has indicated that miR-99a-5p could regulate macrophage accumulation, which was an important event in the development of AS, and alleviate atherosclerotic lesions [22]. This suggested that miR-99a-5p might serve as the potential therapy for AS.

Taken together, our study is aimed at exploring the possibility of miR-99a-5p as a potential therapy for AS. Specifically, we suspected that miR-99a-5p might reduce AS by inhibiting NLRP3 inflammasome activation and promoting macrophage autophagy. In parallel, explaining the specific molecular mechanism was required.

## 2. Materials and Methods

### 2.1. Cell Culture and Preparation

THP-1 monocytes were obtained from the Cell Bank of the Chinese Academy of Sciences (Shanghai, China). The cells were cultured in Roswell Park Memorial Institute (RPMI)-1640 medium (Gibco) containing 10% fetal bovine serum (CC-3202, LONZA) and 1% penicillin/streptomycin solution (Invitrogen) under a 5% CO_2_/95% air atmosphere at 37°C. Phorbol 12-myristate 13-acetate (PMA; Sigma-Aldrich, St. Louis, USA) was applied to induce the differentiation of THP-1 monocytes into macrophages. THP-1 monocytes (1 × 10^6^ cells) were differentiated into macrophages in 6-well plates with 2 mL complete medium containing 100 ng/mL PMA for 48 h. The differentiated macrophages were then treated with 50 g/mL ox-LDL for 24 h. Ox-LDL-induced THP-1 macrophages are used for subsequent determination of TC, TG, and LDL-C, cell viability assay, and RT-qPCR of miR-99a-5p.

### 2.2. Determination of TC, TG, and LDL-C

The supernatants after ox-LDL-induced THP-1 macrophage lysis were collected in 1.5 mL tubes and then washed 3 times with PBS. Following, total cholesterol (TC), triglycerides (TG), and low-density lipoprotein (LDL) were detected by an automatic biochemical analyzer (Hitachi, Tokyo, Japan) using a commercial kit (Beijing North Institute of biological technology, Beijing, China).

### 2.3. Cell Viability Assay

The ox-LDL-induced THP-1 macrophages were seeded in 96-well plates and maintained at 37°C plus 5% CO_2_ for 4 days. At the indicated time (0 h, 12 h, 24 h, 36 h, 48 h, 60 h, and 72 h), 10 *μ*L of CCK-8 reagent (GLPBIO, Montclair, CA, USA) was added to each well, and the plate was incubated for 1-2 h. Then, the cell viability was measured with optical density (OD) absorbance at the wavelength of 450 nm.

### 2.4. Animal Models and Treatment

All animal experiments were approved by the Laboratory Animal Ethical Committee, Hebei Medical University First Affiliated Hospital. Fourteen male apolipoprotein E knockout mice (ApoE^−/−^) and six male C57BL/6J wide-type mice aged 8 weeks and weighing 20–25 g were obtained from the Experimental Animal Center of Beijing University of Medical Sciences (Beijing, China). All mice were housed in a specific pathogen-free (SPF) condition, temperature (20−24°C), humidity-controlled environment (60 ± 5% humidity) with a standard diet, and a 12-hour light/dark cycle.

After acclimatization for 1 week, all ApoE^−/−^ mice were randomly divided into 2 groups: NC mimic group and the miR-99a-5p mimic group. Wide-type mice in the control group were fed a standard rodent diet for 12 weeks. All ApoE^−/−^ mice were fed a high-fat diet (HFD) (1.25% cholesterol, 15.8% fat) for 12 weeks to induce AS. Then, ApoE^−/−^ mice in the NC mimic group and the miR-99a-5p mimic group received injections of NC mimics and miR-99a-5p mimics (20 mg/kg), respectively, through tail veins twice a week for three weeks. During the 3-week treatment period, the diets of mice in each group were the same as before. After that, all mice were sacrificed under anesthesia and intraperitoneally injected with sodium pentobarbital (250 mg/kg), and the intact aortas were harvested for subsequent histopathological evaluation (hematoxylin-eosin, oil red O, Sirius red staining, and immunofluorescence assay) of the atherosclerotic lesion and immunofluorescence (IF) assay.

### 2.5. Determination of Atherosclerotic Lesion

Histopathological evaluation of the aortic valves was performed using hematoxylin-eosin (H&E), oil red O, and Sirius red staining. After the mouse aortic valves were fixed with 4% paraformaldehyde, the tissues were dehydrated with 20% and 30% sucrose solutions, respectively, and then soaked in the bottom of the solution, indicating complete dehydration. Subsequently, the tissues were embedded in an optimal cutting temperature (OCT) compound, and a freezing microtome (Leica, Solms, Germany) was used to cut the tissues into 10 *μ*m slices. Serial sections were stained with several staining solutions following the manufacturer's instructions. The images were taken under a light microscope (Olympus, Tokyo, Japan) and then analyzed using Image Pro-Plus software.

### 2.6. Immunofluorescence (IF) Assay

The slices were firstly deparaffinized in xylene and rehydrated with graded ethanol (90%, 85%, 70%, and 50%). After boiling the sections in citrated buffer (pH 6.0) for 30 minutes, sections were incubated with 3% H_2_O_2_ for 30 minutes and blocked with 5% goat serum for 1 h. After pretreatment, the sections were incubated with anti-CD68 (1 : 50 dilution, Santa Cruz, Dallas, Texas, USA) overnight at 4°C. The sections were then probed with secondary antibody (1 : 200 dilution, Beyotime). Nuclear DNA were counterstained with 4′,6-diamino-2-phenylindole (DAPI) for 10 minutes. The staining was finally observed under a fluorescence microscope (Olympus) at a magnification of 400 times.

### 2.7. Isolation of Peritoneal Macrophages and Cell Culture

The experimental mice were injected intraperitoneally with 2 mL of 3% thioglycollate (Difco) on day 1. After administration, the mice were kept under daily observation to check for abnormal activities. Mice were sacrificed 4 days later, and the peritoneal exudate cells were harvested. After washing twice with cold phosphate-buffered saline (PBS), the peritoneal cells were centrifuged for 10 minutes and resuspended, then seeded into cell culture dishes. The suspension cells were discarded by washing with PBS after 2 h. The purity of macrophages was detected by morphological identification and non-specific esterase staining. Cell viability was determined with trypan blue exclusion. According to these criteria, more than 95% of the adherent cells were viable macrophages. Then, the peritoneal macrophages were performed to detect ELISA of inflammatory cytokines (IL-6, TNF-*α*, and MCP-1) and IL-10 and western blot of related proteins.

### 2.8. Cell Transfection

A total of 4 × 10^5^ peritoneal macrophages were seeded into 6-well plates and cultured to 90%-95% confluence. The macrophages were transfected with the complex of Lipofectamine 2000 (Invitrogen, Thermo Fisher Scientific, USA) and the miR-99a-5p mimics, NC mimics, miR-99a-5p mimics inhibitor NC, miR-99a-5p mimics inhibitor at 50 nM (the mixture ratio was 1 : 1). Six hours later, the overexpression and inhibition of target proteins were confirmed by RT-qPCR. Then, the cells were cultured in a fresh, normal complete medium containing 50 g/mL ox-LDL. All miRNAs were synthesized by RiboBio (Guangzhou, China).

### 2.9. Enzyme-Linked Immunosorbent Assay (ELISA)

ELISA assay was carried out to identify the levels of inflammatory cytokines (IL-6, TNF-*α*, and MCP-1) and IL-10 in the cell supernatants of lytic peritoneal macrophages after transfection. The levels of IL-6, TNF-*α*, MCP-1, and IL-10 were measured using the commercially available ELISA assay kit (Excell Biology, Inc.), following the manufacturer's instructions.

### 2.10. Quantitative Reverse Transcription PCR (RT-qPCR)

The total RNA was extracted from the THP-1 macrophages to detect the miR-99a-5p expression and the peritoneal macrophages to determine the mTOR expression using the TRIzol reagent (Life Technologies), respectively, according to the manufacturer's instructions. The RNA samples were converted into cDNA. Then, the RT-qPCR was conducted using SYBR Green (Solarbio) on a real-time PCR system (ABI, Foster City, CA, USA). Glyceraldehyde 3-phosphate dehydrogenase (GAPDH) and U6 were used as the normalized endogenous controls for mTOR and miR-99a-5p expression, respectively. The relative expression of mRNA was calculated by the comparative cycle threshold (CT) (2^−ΔΔCT^) method. The sequences of primers are shown in Table 1.

### 2.11. Western Blot

The peritoneal macrophages were suspended in RIPA lysis buffer (KeyGen Biotech, Nanjing, China) supplemented with the protease inhibitors cocktail (Roche) for 30 min to extract the total protein. The protein concentration of the supernatants was quantified using the BCA Protein Assay Kit (Solarbio, Beijing, China). Then, the denatured proteins (20 *μ*g/lane) were separated by sodium dodecyl sulfate-polyacrylamide gel electrophoresis (SDS-PAGE) and electrotransferred to PVDF membranes. After the electrotransfer was completed, whole-cell lysates were blocked with a blocking buffer for an hour. Then, the membrane was incubated with primary antibody at 4°C overnight, followed by incubation with appropriate HRP-conjugated secondary antibody. The bands were detected with enhanced chemiluminescence and quantified subsequently to normalization with the density of *β*-actin using ImageJ software (National Institutes of Health, USA).

### 2.12. Bioinformatics Analysis

Target genes of miR-99a-5p were predicted by MIRDB (http://mirdb.org/), TargetScan (version 5.1; http://www.targetscan.org/), PicTar (http://pictar.mdc-berlin.de/), and Miranda (http://www.microrna.org/).

### 2.13. Luciferase Reporter Assay

MTOR mutant-type (MUT) was obtained from mTOR wild-type (WT) by point mutation technique. MTOR WT and MUT sequences were cloned into the pmirGLO vector to construct pmirGLO-mTOR-3′ UTR-WT and pmirGLO-mTOR-3′ UTR-MUT, respectively. Afterwards, HEK 293T cells were transiently cotransfected with pmirGLO-mTOR-3′ UTR-WT or pmirGLO-mTOR-3′ UTR-MUT and NC mimics or miR-99a-5p mimics using Lipofectamine 2000 (Invitrogen, Thermo Fisher Scientific, USA) according to the manufacturer's instructions. After 48 h transfection, the luciferase reporter assay kit (Promega, Madison, WI, USA) was used to measure luciferase activity in the SpectraMax reader (Molecular Devices, Shanghai, China), according to the manufacturer's instructions.

### 2.14. Statistical Analysis

Data are presented as the mean ± standard error of the mean (SEM). Statistical analysis was performed with GraphPad Prism software (version 8.3.0; GraphPad Software, Inc., La Jolla, CA). The significance of the differences between the two groups was determined by *t*-tests. The statistical differences among three or more groups were evaluated by one-way ANOVA with Tukey's post hoc test. *p* < 0.05 was considered statistically significant.

## 3. Results

### 3.1. MiR-99a-5p Was Downregulated in Ox-LDL-Induced THP-1 Macrophages

**Figure 1:**
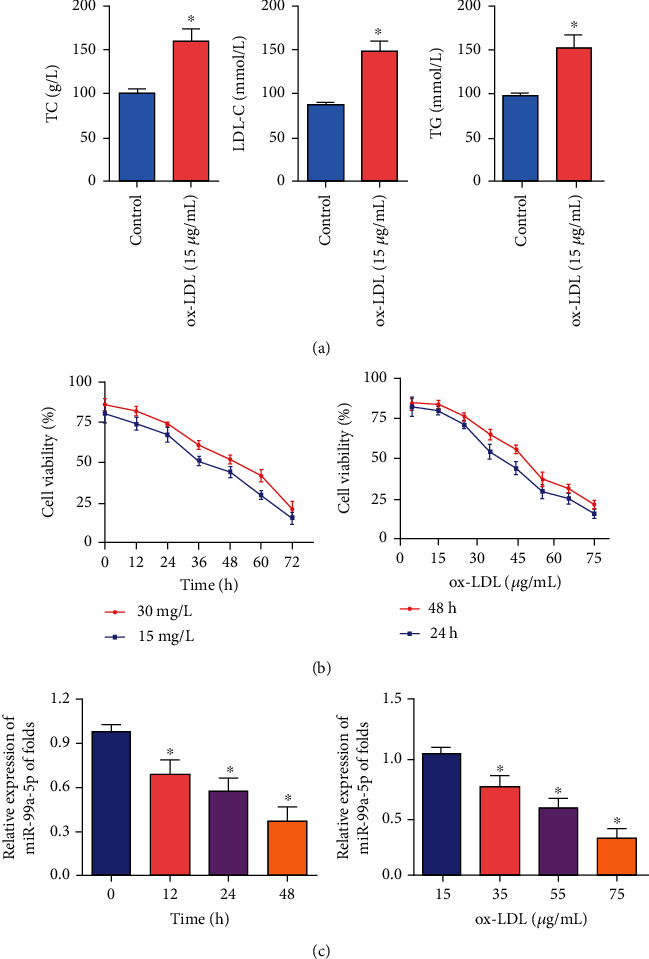
miR-99a-5p was downregulated in ox-LDL-induced THP-1 macrophages. (a) Levels of TC, TG, and LDL-C were increased in the ox-LDL-induced THP-1 macrophages compared with the control, which indicated the formation of foam cells. (b) The cell viability of THP-1 macrophages following ox-LDL treatment was decreased. (a, b) demonstrated that the ox-LDL-induced THP-1 macrophages could be considered as a cell model of atherosclerosis in vitro to detect the aberrant expression of miR-99a-5p. (c) The expression of miR-99a-5p in ox-LDL-induced THP-1 macrophages was downregulated. Data are expressed as mean ± SEM (^∗^*p* < 0.05).

In ox-LDL-induced THP-1 macrophages, TC, TG, and LDL-C were significantly increased compared with the control group, indicating that many lipid droplets have accumulated in the cells after 24 h incubation with 50 g/mL ox-LDL, which showed foam cell formation (the in vitro cell model of AS) (Figure 1(a)). In parallel, cell viability decreased in a time- and concentration-dependent manner in ox-LDL-induced THP-1 macrophages (Figure 1(b)). These data indicated the presence of disease-related characteristics and the formation of the in vitro cell model of AS. Then, we further confirmed that the expression of miR-99a-5p decreased in a time- and concentration-dependent manner in ox-LDL-induced THP-1 macrophages (Figure 1(c)). Overall, the above results suggested that miR-99a-5p was downregulated in ox-LDL-induced THP-1 macrophages and may be involved in the disease progression of AS.

### 3.2. Overexpression of miR-99a-5p Alleviated Atherosclerotic Lesions in the Aortas of ApoE^−/−^ Mice, Which Might Be Related to Inflammation

**Figure 2:**
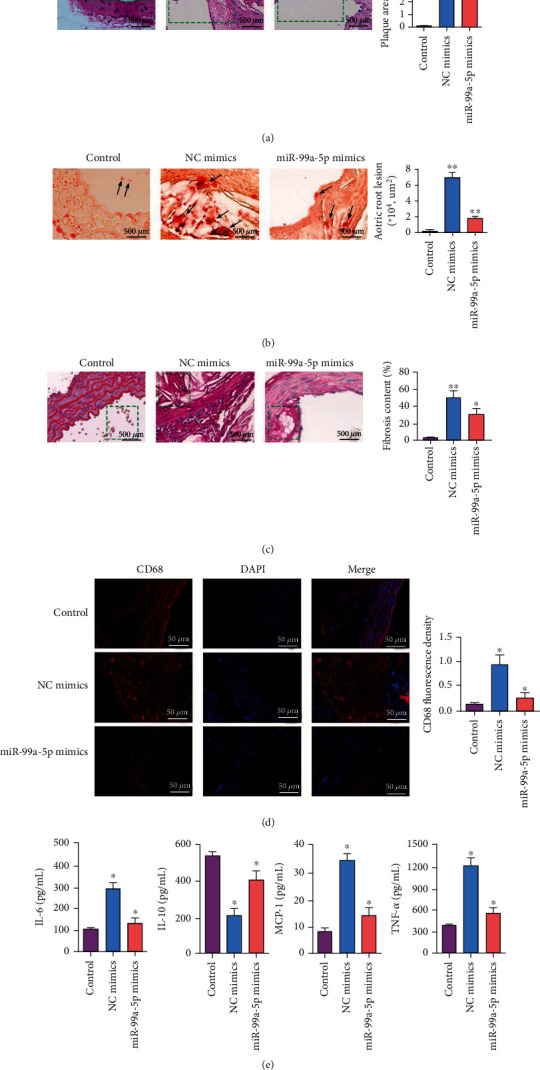
Increased expression of miR-99a-5p could alleviate atherosclerotic lesions in ApoE^−/−^ mice and decrease the secretion of inflammatory cytokines in the peritoneal macrophages. Aorta valves from C57BL/6 mice and ApoE^−/−^ mice injected with NC mimics or miR-99a-5p mimics were stained. (a) Hematoxylin and eosin staining, atherosclerotic lesion area, and the ratio of atherosclerotic lesion area to the total aortic valve (40x, bar = 500 *μ*m). (b) Oil Red O staining and quantitative analysis for the lipid droplet content (40x, bar = 500 *μ*m). (c) Sirius red staining and quantitative analysis for the fibrosis content (40x, bar = 500 *μ*m). (d) Immunofluorescence staining and quantitative analysis for macrophage accumulation by detecting the expression of the macrophage marker CD68 (400x, bar = 50 *μ*m). (e) ELISA results demonstrated that miR-99a-5p could reduce the secretion of inflammatory cytokines (IL-6, MCP-1, and TNF-*α*) and increase the level of IL-10 in the peritoneal macrophages of atherosclerosis. Data are presented as the mean ± SD (^∗^*p* < 0.05; ^∗∗^*p* < 0.01).

The atherosclerotic model was established in ApoE^−/−^ mice to verify the effects of miR-99a-5p on AS in vivo. The plaque areas of the aortas were examined by HE staining. The results indicated that when compared with the control group, the intravascular plaque formation significantly increased in the NC mimic group, while plaques in the miR-99a-5p mimic group showed the opposite trend (Figure 2(a)). Results of oil red O staining showed a significant increase of lipid content in the NC mimic group, while miR-99-5p mimics reduced lipid deposition in the atherosclerotic plaque (Figure 2(b)). Sirius red staining demonstrated that miR-99a-5p mimics could reduce collagen accumulation compared with atherosclerotic mice (Figure 2(c)). Besides, we also investigated whether miR-99a-5p regulated macrophage accumulation, an important event in the progression of AS. Similarly, the obvious macrophage accumulation in atherosclerotic mice and the significant reduction in macrophage content within the lesion with miR-99a-5p were observed by detecting the expression of the macrophage marker CD68 (Figure 2(d)). In addition, the decreased inflammatory cytokines associated with AS (including TNF-*α*, MCP-1, and IL-6) were found with miR-99a-5p mimics, and cytokine synthesis inhibitory factor (CSIF) IL-10 increased (Figure 2(e)). Taken together, these results suggested that increased expression of miR-99a-5p might reduce atherosclerotic lesions, which might be related to inflammation.

### 3.3. Overexpression of miR-99a-5p Might Inhibit Activation of NLRP3 Inflammasome and Then Reduce the Secretion of Inflammatory Cytokines

**Figure 3:**
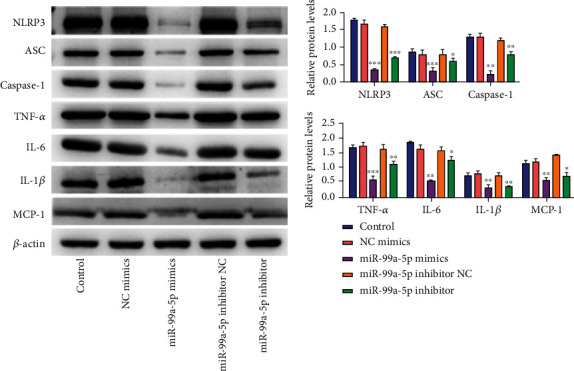
Overexpression of miR-99a-5p inhibited activation of NLRP3 inflammasome and then reduced the secretion of inflammatory cytokines in the peritoneal macrophages of atherosclerosis, while the miR-99a-5p inhibitor showed an opposite trend. Relative protein levels of inflammasome complex (including NLRP3, ASC, and Caspase-1) and inflammatory cytokines (TNF-*α*, IL-6, IL-1*β*, and MCP-1) among the control group, NC mimic group, miR-99a-5p mimic group, miR-99a-5p mimics inhibitor NC group, and miR-99a-5p mimics inhibitor group. The results demonstrated that miR-99a-5p decreased the expression of inflammasome complex (including NLRP3, ASC, and Caspase-1) and inflammatory cytokines (TNF-*α*, IL-6, IL-1*β*, and MCP-1). Data are presented as the mean ± SD (^∗^*p* < 0.05; ^∗∗^*p* < 0.01; ^∗∗∗^*p* < 0.001).

Given the above observations, we further probed whether miR-99a-5p was related to NLRP3 inflammasome activation and following induction of the maturation and secretion of inflammatory cytokines. The results of the Western blot revealed that compared with the NC mimic group, the protein expression of inflammasome complex (including NLRP3, ASC, and Caspase-1) was markedly decreased in the miR-99a-5p mimic group, while enhanced by the miR-99a-5p mimics inhibitor (Figure 3). Moreover, similar results were observed in inflammatory cytokines (TNF-*α*, IL-6, IL-1*β*, and MCP-1). All these data suggested that forced expression of miR-99a-5p might inhibit the activation of the NLRP3 inflammasome and then reduced the secretion of inflammatory cytokines.

### 3.4. MiR-99a-5p Directly Targeted mTOR

**Figure 4:**
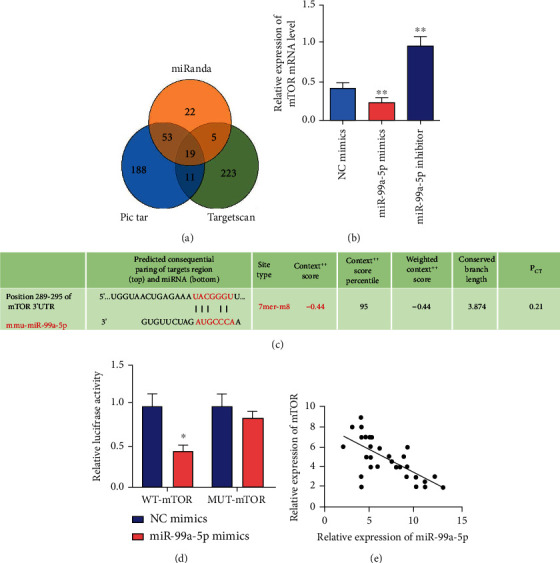
miR-99a-5p directly targeted mTOR. (a) Nineteen targets of miR-99a-5p were identified using the bioinformatics prediction. (b) RT-qPCR results showed that relative mRNA levels of mTOR were downregulated after miR-99a-5p, while the miR-99a-5p group showed an opposite trend. (c) The 3′-UTR of mTOR (position 289-295) was speculated to contain a complementary region of miR-99a-5p seed sequences. (d) Luciferase reporter plasmids containing the WT or MUT 3′-UTR of mTOR were cotransfected with NC mimics or miR-99a-5p mimics into HEK 293T cells. Luciferase assay was performed after 48 h transfection. The results demonstrated that miR-99a-5p could directly target mTOR. (e) MiR-99a-5p negatively regulated mTOR. Data are presented as the mean ± SD (^∗^*p* < 0.05; ^∗∗^*p* < 0.01).

Based on the above results, we then explored the targets of miR-99a-5p. Nineteen targets of miR-99a-5p were identified by using the bioinformatics prediction (Figure 4(a)). Based on previous work [23], mTOR was selected for further study. It was confirmed that the overexpression of miR-99a-5p markedly reduced mTOR mRNA level, which was a negative correlation, while the miR-99a-5p inhibitor showed the opposite trend (Figures 4(b) and 4(e)). The 3′-UTR of mTOR (position 289-295) was speculated to contain a complementary region of miR-99a-5p seed sequences (Figure 4(c)). Results of the dual-luciferase reporter assay exhibited that the cotransfection of miR-99a-5p mimics and mTOR-WT into HEK 293T cells could suppress the relative luciferase activity, while the cotransfection of miR-99a-5p mimics and mTOR-WT-MUT enhanced the trend, which suggested that miR-99a-5p directly targeted mTOR (Figure 4(d)). In general, the data indicated that miR-99a-5p might directly target and negatively regulate mTOR, meanwhile coinvolved in AS disease progression.

### 3.5. MiR-99a-5p Suppressed NLRP3 Inflammasome Activation and Enhanced Macrophage Autophagy by Inhibiting mTOR, Therefore, Ameliorating AS

**Figure 5:**
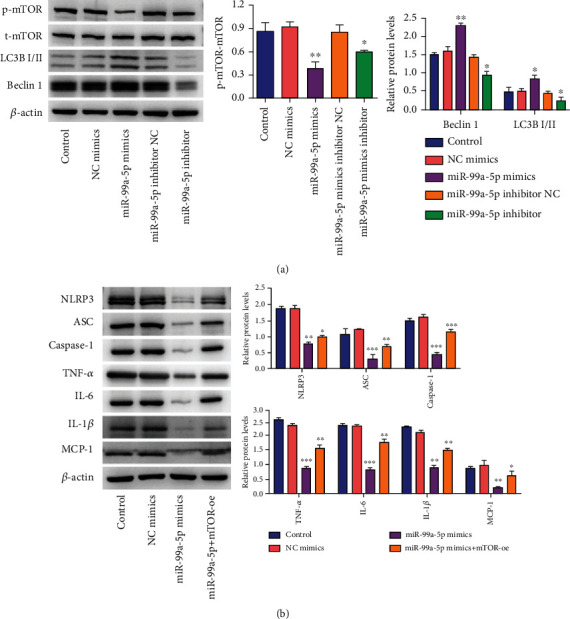
MiR-99a-5p mimics might suppress NLRP3 inflammasome activation and enhance macrophage autophagy by inhibiting mTOR. (a) The expressions of mTOR and autophagy marker protein (Beclin 1 and LC3B) were determined by Western blot assay following miR-99a-5p mimics and its inhibitor. The results demonstrated that miR-99a-5p decreased the expression of mTOR and enhanced macrophage autophagy while miR-99a-5p showed the opposite trend. (b) The expressions of inflammasome complex (NLRP3, ASC, and Caspase 1) and inflammatory cytokines (TNF-*α*, IL-6, IL-1*β*, and MCP-1) after miR-99a-5p mimics and its mTOR-oe. The Western blot demonstrated that miR-99a-5p decreased the expression of inflammasome complex and inflammatory cytokines, while mTOR could reverse the trend. Data are presented as the mean ± SD (^∗^*p* < 0.05; ^∗∗^*p* < 0.01; ^∗∗∗^*p* < 0.001).

We further quantified the regulation of mTOR by miR-99a-5p and explored whether miR-99a-5p could affect autophagy, an important event in AS. The results of Western blot demonstrated that the protein expression of LC3B and Beclin-1 (representative markers monitoring autophagy) of peritoneal macrophages in the miR-99a-5p mimic group was higher than that in the NC mimic group, while the opposite trend of mTOR expression was observed (Figure 5(a)). In parallel, the miR-99a-5p inhibitor could reverse the above trend (Figure 5(a)). Recent evidence supported the interplay between NLRP3 inflammasome and mTOR [24]. The results of the Western blot showed that mTOR overexpression could reverse the decreased trend of NLRP3 inflammasome in the miR-99a-5p mimic group and subsequently enhance the secretion of inflammatory cytokines such as TNF-*α*, IL-6, IL-1*β*, and MCP-1 (Figure 5(b)). Taken together, miR-99a-5p might suppress NLRP3 inflammasome activation and enhance macrophage autophagy by inhibiting mTOR.

## 4. Discussion

**Figure 6:**
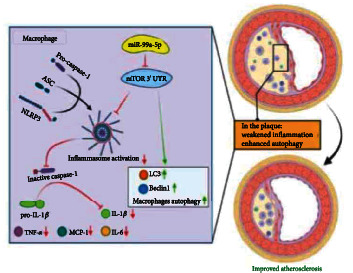
Schematic representation of the mechanism by which miR-99a-5p suppressed NLRP3 inflammasome activation and enhanced macrophage autophagy by targeting mTOR, therefore, regulating atherosclerosis. miR-99a-5p directly targeted mTOR and negatively regulated mTOR expression. In vitro, miR-99a-5p overexpression could inhibit NLRP3 inflammasome activation and enhance macrophage autophagy mediated by mTOR. In vivo, the specific overexpression of miR-99a-5p by intravenous injection of miR-99a-5p mimics significantly abated atherosclerotic lesions formatted in high-fat diet apolipoprotein E knockout mice.

To the best of our knowledge, this was the first study that revealed miR-99a-5p might reduce AS by targeting mTOR. The most striking findings in the present study were as follows: (1) miR-99a-5p was downregulated in ox-LDL-induced THP-1 macrophages; (2) overexpression of miR-99a-5p might inhibit activation of the inflammasome and then reduce the secretion of inflammatory cytokines, therefore, alleviating AS in ApoE^−/−^ mice; (3) miR-99a-5p directly targeted mTOR, then suppressed NLRP3 inflammasome activation and enhanced autophagy of macrophages, thereby improving AS (Figure 6).

miRNAs have various characteristics such as regulating cell growth, proliferation, differentiation, migration, aging, apoptosis, and angiogenesis [25, 26]. Previous studies have indicated that the abnormal expression of miRNAs and dysregulation in functions were closely related to cardiovascular diseases, especially AS [17, 20, 27–30]. Moreover, accumulating studies have been conducted on the potential of miRNA regulating AS [31, 32]. Therefore, it has become a new research hotspot and prognostic and therapeutic tool for AS [33–35]. Previous studies on miR-99a-5p have more focused on cancer [36, 37], but recent research has demonstrated that miR-99a-5p was involved in the progression of AS [22]. Therefore, we first explored the relationships between the two and found that miR-99a-5p expression was downregulated in ox-LDL-induced THP-1 macrophages.

Then, the in vivo role of miR-99a-5p in the AS disease progression has continued to arouse interest. The staining results showed that miR-99a-5p could improve the AS lesions. In addition, increasing attention to the cause of the above trend should be paid. Several previous studies have revealed that the expression level of miR-99a-5p might be related to inflammation and immune responses [38–40]. Since AS was a chronic inflammatory disease [41, 42], we then explored whether miR-99a-5p could affect inflammatory cytokines. Thrillingly, decreased secretion of inflammatory cytokines (including TNF-*α*, IL-6, and MCP-1) and increased anti-inflammatory cytokines (IL-10) were observed with the miR-99a-5p overexpression. This was also consistent with previous research. As is well-known, the maturity and secretion of inflammatory cytokines (IL-1*β*/IL-18) are mediated by the NLRP3 inflammasome composed of ASC, caspase-1, and NLRP3 [43, 44]. Moreover, this was a consensus that NLRP3 inflammasome activation contributed to the vascular inflammatory response driving AS development and progression [6, 15, 45]. In the present study, the protein expression of NLRP3 inflammasome and inflammatory cytokines were markedly decreased under miR-99a-5p overexpression, whereas the miR-99a-5p inhibitor showed the opposite trend. Taken together, it was reasonably suspected that miR-99a-5p may inhibit the activation of NLRP3 inflammasome and following secretion of inflammatory cytokines, thereby alleviating AS. Mechanically, further investigation remained essential.

MTOR regulated biomass accumulation and metabolism by modulating key cellular processes (including protein synthesis and autophagy) [46, 47], and dysregulation of mTOR signaling has been implicated in multiple diseases, such as AS [48, 49]. Furthermore, increasing studies have found that mTOR could interact with NLRP3 inflammasome and miR-99a-5p, respectively [23, 24, 50–52]. However, accumulating studies have indicated that the same microRNA played an essential role in the treatment of various diseases by interacting with different pathways [53]. Therefore, it remained necessary to predict the target genes of miR-99a-5p in AS using bioinformatics analysis in the present study. By the bioinformatics prediction, we found mTOR was one of the miR-99a-5p targets, and biological processes were mainly involved in the inflammatory response, mTOR signaling. In addition, the correlation analysis and luciferase reporter assay further confirmed that miR-99a-5p directly targeted and negatively regulated mTOR. Based on the above research and results, mTOR was finally selected as a potential target for subsequent studies. Then, results of the Western blot showed that miR-99a-5p indeed inhibited mTOR expression, while miR-99a-5p inhibitor exhibited an opposite trend. In parallel, consistent with speculation, miR-99a-5p inhibited NLRP3 inflammasome activation and secretion of inflammatory cytokines, while the addition of mTOR reversed the situation. Thereby, it was reasonably concluded that miR-99a-5p might inhibit NLRP3 inflammasome activation and following secretion of inflammatory cytokines via restraining mTOR.

Recently, numerous studies have proved that macrophage autophagy played an important role in the development of AS, that is, macrophages are the key catabolic workhorse specialized in clearing lipid and dead cell debris in the atherosclerotic plaque [16, 54, 55]. Furthermore, the significance of miRNAs in autophagy has been deeply studied in AS [56], and in parallel, the regulation of miR-99a-5p and autophagy has been implicated in several cancers [18]. Given the above research, we here explored whether miR-99a-5p could regulate macrophage autophagy and reduce macrophage accumulation, which were vital events in the development of AS. As expected, results showed a remarkable increased expression of autophagy marker (LC3B-II/I and Beclin1) and a significant decrease in macrophage accumulation after miR-99a-5p, suggesting the activation of macrophage autophagy. Furthermore, the opposite trend with miR-99a-5p inhibitor supported the finding in another way.

Taken together, the present study demonstrated that miR-99a-5p could inhibit NLRP3 inflammasome activation and enhance macrophage autophagy by constraining mTOR, then improving AS. The findings also suggested that miR-99a-5p may be a potential therapeutic strategy for AS. Nevertheless, these findings were drawn from the mouse model, which was not enough to translate into the complex framework of AS in humans. Besides, it was well-known that mTOR negatively regulated the macrophage autophagy and then affected the stability of atherosclerotic plaques [57–59]. However, the detailed exploration of how MTOR regulated autophagy in the present study was lacking. Moreover, mTOR was one of the numerous target genes of miR-99a-5p. Other genes regulated by miR-99a-5p may be also involved in the progression of AS. Thus, more specific molecular mechanisms underlying the involvement of miR-99a-5p and its targets in AS remained to be further explored.

## 5. Conclusion

In brief, the present study revealed that miR-99a-5p might suppress NLRP3 inflammasome activation and induce macrophage autophagy by inhibiting mTOR, then alleviating AS. Thereby, it is reasonably expected that miR-99a-5p may be a potential therapy for AS, but efforts are still needed to provide more evidence.

## Figures and Tables

**Table 1:** Primers for RT-qPCR.

Gene name		Primer sequence
mTOR	Forward primer (5′-3′)	TCCGAGAGATGAGTCAAGAGG
	Reverse primer (5′-3′)	CACCTTCCACTCCTATGAGGC
U6	Forward primer (5′-3′)	GTAGATACTGCAGTACG
	Reverse primer (5′-3′)	ATCGCATGACGTACCTGAGC
miR-99a-5p	Forward primer (5′-3′)	TGGCATAAACCCGTAGATCC
	Reverse primer (5′-3′)	CCATAGAAGCGAGCTTGTG
GAPDH	Forward primer (5′-3′)	CGCTCTCTG CTCCTCCTGTTC
	Reverse primer (5′-3′)	ATCCGTTGACTCCGACCTTCAC

Note: miR-99a-5p: microRNA-99a-5p; mTOR: mammalian target of rapamycin.

## Data Availability

The datasets analyzed during the current study are available from the corresponding author upon reasonable request.
